# Genome-Wide Identification, Evolutionary Expansion, and Expression Analyses of Aux/IAA Gene Family in *Castanea mollissima* During Seed Kernel Development

**DOI:** 10.3390/biology14070806

**Published:** 2025-07-03

**Authors:** Yujuan Tian, Jingmiao Huang, Jinxin Wang, Dongsheng Wang, Ruimin Huang, Xia Liu, Haie Zhang, Jingzheng Zhang, Xiangyu Wang, Liyang Yu

**Affiliations:** 1 Engineering Research Center of Chestnut Industry Technology, Ministry of Education, Hebei Normal University of Science and Technology, Qinhuangdao 066004, China; 19565399593@163.com (Y.T.); wdsgfly@126.com (D.W.); rmhuang@163.com (R.H.); zhang33haie4@163.com (H.Z.); zhangjingzheng@126.com (J.Z.); 2 Shijiazhuang Institute of Pomology, Hebei Academy of Agriculture and Forestry Sciences, Shijiazhuang 050061, China; huangjm94@126.com (J.H.); wangjinxin1948@163.com (J.W.); 3 College of Smart Agriculture, Chongqing University of Arts and Sciences, Chongqing 402160, China; liuxiavip8@163.com; 4 The Office of Scientific Research, Hebei Normal University of Science and Technology, Qinhuangdao 066004, China

**Keywords:** Aux/IAA gene family, *Castanea mollissima*, developmental stage, RNA-seq, seed size, plant hormone

## Abstract

Auxin/induced-3-acetic acid (Aux/IAA) serves as a key regulator in the auxin signaling pathway of plants, which exhibits crucial functions in the development of plants. However, the Aux/IAA gene family has not yet been characterized in the genome of *Castanea mollissima*, an important food source in the Northern Hemisphere. During this research, 23 *Aux*/*IAA* genes were identified in the *C. mollissima* genome, which were unevenly distributed across seven chromosomes. *CmAux*/*IAA* genes were assigned to four subfamilies by phylogenetic analysis, and members of the same subfamily exhibited similar molecular characteristics. Collinear analysis revealed that the expansion of *CmAux*/*IAA* genes was primarily driven by whole-genome duplication (WGD) and purifying selection. The promoter regions of *CmAux*/*IAA* genes were enriched with development-related and hormone-related *cis*-acting elements, suggesting their crucial functions in the growth and hormonal regulation of *C. mollissima*. Upon the maturation of the seed kernels, the size and starch content exhibited a significant increasing trend, alongside notable changes in hormone levels. Given the connections between expression levels and physiological indicators, as well as weighted gene co-expression network analysis (WGCNA) analysis, *CmIAA27a*, *CmIAA27b*, and *CmIAA27c* were identified as potential regulators involved in the development of *C. mollissima* seed kernels. Furthermore, the reliability of the transcriptomic data was further confirmed by RT-qPCR experiments. Overall, this study provides a theoretical basis for the evolutionary expansion of the Aux/IAA gene family in *C. mollissima*, alongside its potential functions in seed kernel development.

## Simple Summary

The low yield per unit area and unstable seed kernel quality severely limit the development of the Chinese chestnut (*Castanea mollissima*) industry, and seed kernel size and starch content are important factors affecting Chinese chestnut yield and quality. *Aux*/*IAA* genes, as one of the important gene families involved in plant seed kernel growth and development, play a crucial role in seed morphogenesis and nutrient accumulation. Therefore, a systematic characterization of the Aux/IAA gene family in the Chinese chestnut genome was conducted. A total of 23 Aux/IAA members were identified in the Chinese chestnut genome and classified into four groups (Group I–IV). Specifically, this study systematically demonstrated that *Aux*/*IAA* genes in Chinese chestnut actively participate in plant growth and development. To understand the developmental characteristics of Chinese chestnut seed kernels, morphological indices, starch content, and hormone levels were measured during five post-anthesis stages. To further elucidate the role of *Aux*/*IAA* genes in seed kernel development, RNA-seq analysis was performed. Correlation analysis and WGCNA were combined with growth indicators to screen candidate genes potentially influencing Chinese chestnut seed kernel development, providing insights into their potential functions in plant fruit development.

## 1. Introduction

Auxin exhibits crucial functions during the development of plants based on the regulation of cell elongation, apical dominance, root development, tropic responses, fruit development, organ abscission, vascular tissue differentiation, and stress responses [1]. Aux/IAA is one of the major gene families that participate in early auxin responses, and it has been regarded as one of the most dynamic and crucial gene regulatory systems [2]. Furthermore, Aux/IAA could serve as key components of the auxin signaling pathway, which could modulate the auxin-responsive genes by specifically interacting with the auxin response factor (*ARF*), thereby participating in physiological processes (such as plant growth, signal transduction, and metabolism) [2]. The typical Aux/IAA contains four highly conserved domains. Domain I was identified to harbor the conserved EAR repression motif (LxLxLx), which was known to interact with ethylene response factors (ERFs), and the transcriptional corepressor TOPLESS (TPL) bound to repress the expression of downstream auxin-responsive genes based on this mechanism [3]. Domain II contains a conserved “GWPPV” motif, which is regulated by transport inhibitor response 1 (TIR1), and it could primarily determine the protein stability of Aux/IAA proteins [4]. Domains III and IV constitute the ARF-interacting regions, and they share homology with the C-terminal dimerization domains (CTDs) of ARF proteins. Functionally, these domains synergistically facilitate both homomeric and heteromeric dimerization between Aux/IAA and ARF proteins, thereby modulating the auxin signal transduction [5,6]. Notably, the Aux/IAA gene family exhibits diversity with the aspects of degradation and auxin responsiveness at the molecular regulatory level, and these characteristics were dependent on the “GWPPV” motif in Domain II [1]. Additionally, Aux/IAA could mediate auxin responses through dual signaling pathways. The typical Aux/IAA predominantly depends on the molecular recognition properties of Domain II, which could interact with TIR1/AFB receptors, and an Aux/IAA-AUXIN-TIR1/AFB ternary complex would be formed upon auxin binding [7]. Subsequently, this complex integrates with the C-terminal domain of F-box proteins into the ubiquitin-protein ligase complex (SCF), ultimately assembling into a functional SCF^TIR1^-AUXIN-Aux/IAA complex [8]. The SCF complex could catalyze the ubiquitination of Aux/IAA proteins when intracellular auxin concentrations reach a threshold, thus promoting the proteasomal degradation of 26S, and the inhibited ARF transcription factors were released to activate downstream target genes [8]. Alternatively, atypical Aux/IAA proteins with the absence of Domain II could transduce auxin signals through the TMK1-IAA32/34-ARF pathway [9]. Overall, the discovery of this pathway reveals a TIR1/AFB receptor-independent regulatory paradigm, which could significantly expand the understanding of the auxin signaling networks.

Functionally, the Aux/IAA gene family exhibits both tissue specificity and functional diversity. In *Arabidopsis thaliana*, *IAA3*/*SHY2* exhibits dual regulatory functions in early auxin-responsive gene expression, which could serve as both a negative and positive regulator. Notably, ectopic GUS expression could be observed in hypocotyls, cotyledons, petioles, and root vascular tissues under auxin-deficient conditions [2]. In *Populus*, *PtoIAA9* and *PtoARF5* could interact with each other in an auxin-dependent manner, which can regulate the development and secondary xylem formation of secondary vascular tissues during wood development [10]. Remarkably, over-expression of *MdIAA26* in *Malus pumila* calli and *A. thaliana* could contribute to the enhancement of anthocyanin accumulation, alongside the up-regulation of key anthocyanin biosynthesis-related genes [11]. Recently, the regulatory functions of *Aux*/*IAA* genes in fruit development have been highlighted by more studies. For instance, down-regulation of *SlIAA6* expression in *Solanum lycopersicum* could trigger fruit set after ovule fertilization [12]. In *A. thaliana*, the over-expression of *IAA1* with a Domain II mutation could induce damage to cell elongation and cell division in inflorescences [13]. Regarding *Musa nana*, *MaIAA17*-like could modulate the fruit ripening based on the transcriptional up-regulation of the key genes connected with fruit softening and de-greening [14]. Similarly, in *Fragaria ananassa*, both *FaAux/IAA* and *FaAux*/*IAA2* participated in the early development of fruits, where the increased transcript levels might contribute to the growth of auxin-mediated fruit growth and the delay of *F. ananassa* ripening [15]. Members of the Aux/IAA gene family have been identified in various plant species, including *A. thaliana* (29) [16], *Oryza sativa* (31) [17], *S. lycopersicum* (26) [18], *Populus* (35) [2], *M. pumila* (33) [19], *Zea mays* (40) [20]. The members of the Aux/IAA gene family in different plants are classified into varying numbers of groups. For instance, 26 Aux/IAA members in *S. lycopersicum* are divided into two clades [21]. In *O. sativa*, 31 Aux/IAA members are categorized into six subfamilies (A1, A2, A3, B1, B2, and B3) [22], while 31 Aux/IAA members in *Z. mays* are grouped into seven subfamilies [20]. Actually, the diversity of Aux/IAA protein sequences across different plants leads to varying results in phylogenetic analysis, which may stem from the evolutionary pressure exerted by diverse environments on the formation of *Aux*/*IAA* genes in various plant species. Notably, the Aux/IAA gene family exhibits distinct evolutionary patterns across different plant species, alongside the remarkable functional diversification and specificity, which likely reflects the adaptations of these plants to different environmental and ecological conditions [1].

*C. mollissima* has gained more attention globally due to the fact that its seed kernels are rich in starch, protein, and essential micronutrients [23]. Furthermore, the low caloric content and high protein levels of *C. mollissima* make it an excellent plant-based protein source for human nutrition [24,25]. Given the pivotal functions of *Aux*/*IAA* genes in seed morphogenesis and nutrient accumulation, the systematic characterization could provide valuable insights into the genetic regulation of the further development of seed kernels. Overall, the first comprehensive identification and bioinformatics analysis of *Aux*/*IAA* genes in *C. mollissima* was presented. Furthermore, based on the integrated analysis of morphological characteristics, phytohormone profiles, and gene expression patterns across different developmental stages of *C. mollissima* seed kernels, it was proposed that *CmIAA27a*, *CmIAA27b*, and *CmIAA27c* might exhibit crucial functions during the development of seed kernels. These findings construct a basis for the exploration of the mechanisms by which *Aux*/*IAA* genes regulate the growth of seed kernels.

## 2. Materials and Methods

### 2.1. Identification, Molecular Characteristics, and Phylogenetic Analysis

The *C. mollissima* genome data were retrieved from the Castanea Genome Database (http://castaneadb.net/#/) (accessed on 10 January 2025). With the application of 29 *A. thaliana* Aux/IAA protein sequences as reference [16], the BLAST v.2.2.30+ searches were performed against all *C. mollissima* protein sequences [26]. For further analysis, HMMER 3.0 software was employed with the *Aux*/*IAA* Hidden Markov Model (PF02309) obtained from the Pfam database (https://www.ebi.ac.uk/interpro/entry/pfam/#table) (accessed on 10 January 2025) [27]. Subsequently, all candidate sequences were submitted to Batch-CDD (https://www.ncbi.nlm.nih.gov/Structure/bwrpsb/bwrpsb.cgi) (accessed on 10 January 2025) to verify the existence of Aux/IAA conserved domains, and 23 *CmAux*/*IAA* genes were ultimately identified for further analysis (Table S1). Protein physicochemical properties and conserved motif arrangements were analyzed using ExPASy (https://www.expasy.org/) (accessed on 10 January 2025) and MEME suite (https://meme-suite.org/meme/) (accessed on 10 January 2025), respectively. Subcellular localization and *cis*-acting elements were predicted by WoLF-PSORT (https://wolfpsort.hgc.jp/) (accessed on 10 January 2025) and PlantCARE (https://bioinformatics.psb.ugent.be/webtools/plantcare/html/) (accessed on 10 January 2025), respectively. Additionally, the gene structure information was obtained from *C. mollissima* GFF3 files and visualized using TBtools v. 2.313 [28]. Aux/IAA protein sequences from *A. thaliana* and *O. sativa* were acquired from The Arabidopsis Information Resource (TAIR) (https://www.arabidopsis.org/) (accessed on 10 January 2025) and Rice Genome Annotation Project (RGAP) databases (https://rice.uga.edu/) (accessed on 10 January 2025) (Table S2), respectively. MEGA 7.0 was employed for phylogenetic analysis with the maximum likelihood method. The *CmAux*/*IAA* genes were systematically classified and renamed based on sequence similarity with their *A. thaliana* counterparts [29].

### 2.2. Chromosomal Distribution and Collinear Analysis

Chromosomal localization information of *CmAux*/*IAA* genes was extracted from the *C. mollissima* GFF3 file and visualized by TBtools v.2.313 [28]. Genome data of *A. thaliana*, *Quercus robur*, *Vitis vinifera*, *O. sativa*, and *Z. mays* were obtained from the Phytozome database (https://phytozome-next.jgi.doe.gov/) (accessed on 10 January 2025). Furthermore, MCScanX v1.0 software was employed to investigate the collinear relationships between these plant genomes and the *C. mollissima* genome [30]. Based on intra-genomic collinear analysis, the duplication types of *CmAux*/*IAA* genes were determined by the examination of homologous gene dot-plot in collinear gene pairs and calculation of synonymous substitution rates (Ks) values in collinear blocks according to the previously established methodology [31].

### 2.3. GO/KEGG Enrichment, Transcription Factors (TFs) Regulatory and Protein-Protein Interaction (PPI) Network Analysis

Functional annotation of *C. mollissima* genes was performed by eggNOG-mapper for GO and KEGG annotation [32], followed by GO/KEGG enrichment analysis and visualization with TBtools v.2.313 [28]. The Plant Transcriptional Regulatory Map (PTRM) (https://plantregmap.gao-lab.org/index-chinese.php) (accessed on 20 January 2025) was employed for the prediction of transcription factors binding to the 2000 bp upstream regions of *CmAux*/*IAA* genes (*p*-value ≤ 1 × 10^−5^), and the word clouds were generated based on the ggplot2 package [33]. The STRING database (https://cn.string-db.org/) (accessed on 20 January 2025) was employed to investigate the protein-protein interaction networks among CmAux/IAA proteins [34,35]. Additionally, Cytoscape software (v3.9.1) was employed for the visualization of the predicted transcriptional regulatory networks and protein interaction networks [36].

### 2.4. Plant Materials and Phenotypic Determination

The *C. mollissima* cultivar ‘Liuyuebao’ has been widely cultivated in China for its large kernel size and superior quality. In this study, five-year-old ‘Liuyuebao’ trees were used as plant materials, which were grown at the Experimental Station of Hebei Normal University of Science & Technology in Qinhuangdao City, Hebei Province (39°66′ N, 119°22′ E). Specifically, the samples were collected at 60, 70, 80, 90, and 100 days after flowering (designated as T1 to T5 stages, respectively). Digital calipers were employed to measure the morphological parameters (including kernel width, length, and thickness), while fresh weight was determined with an electronic balance. For biochemical analyses, starch content was quantified by the dual-wavelength method, and phytohormone levels were measured by the LC-MS/MS platform. Through the above methods, the starch and hormone contents in samples of the same mass were measured.

### 2.5. Expression Analysis of CmAux/IAA Genes

We obtained the 127 runs across six projects from the SRA database to analyze the expression patterns of *CmAux*/*IAA* genes in different tissues and under three types of environmental stress (Table S3). Specifically, different tissues include buds (three developmental stages), ovules (fertile and abortive ovules at three developmental stages), flowers (male flowers: first and secondary flowers, female flowers: first and secondary flowers), seed kernels (five developmental stages of two *C. mollissima* cultivars ‘Yanshanzaofeng’ and ‘Yanlong’). Three types of abiotic stress include drought (five varying degrees of drought across two varieties, ‘Yanshanzaofeng’ and ‘Dabanhong’), cold, and heat stresses (four different degrees of cold stress and heat stress, respectively). The raw RNA-seq data in SRA format were processed using Sratoolkit 3.0 for conversion to Fastq format [37]. For data visualization, expression values were normalized via the scale method in TBtools and subsequently log2-transformed (log_2_(FPKM+1)), with the results presented as a heatmap depicting relative expression levels [28].

### 2.6. RNA Sequencing and DEG Analysis

The RNAprep Pure Plant Kit (Tiengen, Beijing, China) was employed for the extraction of total RNA, while the RNA purity was analyzed using either a Qubit 4.0 Fluorometer or an MD microplate reader. For transcriptome sequencing, RNA was reverse transcribed into cDNA by the NEBNext^®^ Ultra™ RNA Library Prep Kit for Illumina (San Diego, CA, USA), followed by sequencing on the NovaSeq 6000 platform (Illumina). Notably, all RNA-seq data were deposited in the NCBI database (BioProject number: PRJNA1269733). The published N-11 *C. mollissima* genome served as the reference genome, and the filtered clean reads were aligned to the reference genome by HISAT2 v2.0.5 [38]. FeatureCounts was employed for the quantification of the expression as FPKM values [39], and differentially expressed genes (DEGs) were analyzed using DESeq2 v1.4.5 [40].

### 2.7. WGCNA Analysis and RT-qPCR Validation

The MetWare Cloud platform was employed in this research (https://cloud.metware.cn/#/tools/detail?id=247) (accessed on 10 March 2025) for the WGCNA analysis, with a merging threshold of 0.25 for module dendrogram pruning and a correlation coefficient cutoff of 0.6 for module-sample heatmap visualization. Additionally, gene-specific primers were designed by Primer Premier 5 and synthesized by MetWare Biotechnology Co., Ltd. (Wuhan, Hubei Province, China). (Table S4) [41]. The Evo M-MLV RT Mix Kit was employed for quantitative real-time PCR (RT-qPCR), with gDNA Clean for qPCR Ver.2 and 2 × SG Green qPCR Mix on a Bio-Rad CFX Manager system. The *18S* gene of *C. mollissima* was used as the reference, and the 2^−ΔΔCt^ method was used to analyze the relative gene expression levels [42].

### 2.8. Statistical Analysis

Data analysis was performed using GraphPad Prism 9.5, focusing on variance analysis [43], with means compared via the least significant difference (LSD) test. The relationships between variables were examined using Pearson correlation analysis in SPSS 26.0 (IBM Corp., Armonk, NY, USA), with statistical significance set at *p* < 0.05 [44]. All statistical assessments were based on three replicate samples.

## 3. Results

### 3.1. Identification and Phylogenetic Analysis

**Figure 1:**
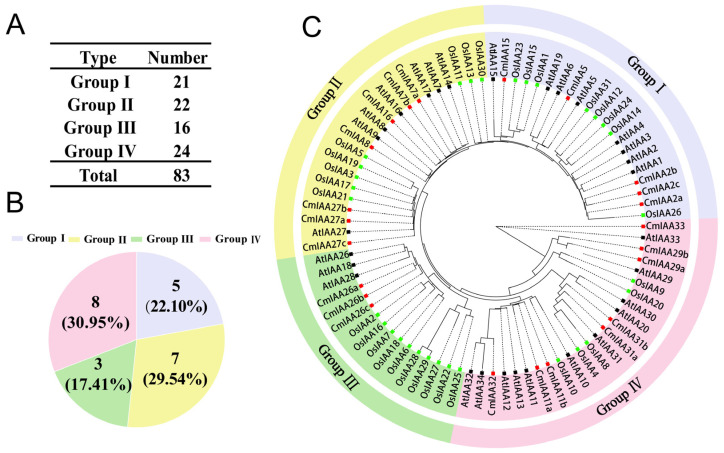
Phylogenetic analysis of the Aux/IAA genes of *C. mollissima*, *A. thaliana*, and *O. sativa*. (**A**) Population distribution of 83 Aux/IAA gene family members from *C. mollissima*, *A. thaliana*, and *O. sativa* in four subfamilies. (**B**) The proportion of the *Aux*/*IAA* genes in *C. mollissima* across four subfamilies. (**C**) Phylogenetic evolution of *Aux*/*IAA* members of *C. mollissima* (23), *A. thaliana* (29), and *O. sativa* (31). The outer circles in purple, yellow, green, and pink colors correspond to the four subfamilies Group Ⅰ, Group Ⅱ, Group Ⅲ, and Group Ⅳ. The inner green, black, and red squares represent the Aux/IAA members from *O. sativa*, *A. thaliana*, and *C. mollissima*.

According to the results, 23 *Aux*/*IAA* genes were identified, which exhibited diverse physicochemical properties. For instance, the CmAux/IAA proteins varied in length from 169 (*CmIAA31b*) to 382 (*CmIAA8*) amino acids, with molecular weights from 18.71 (*CmIAA33*) to 41.29 kDa (*CmIAA8*). Furthermore, their aliphatic index values spanned from 62.22 (*CmIAA7a*) to 91.18 (*CmIAA33*). Notably, the total mean values for the hydrophilicity of the CmAux/IAA proteins were all less than zero, which could indicate that all these proteins were hydrophilic. Furthermore, most proteins were predicted to be alkaline, with theoretical isoelectric points (pI) greater than seven. Except for the stable proteins (including *CmIAA11a*, *CmIAA29b*, *CmIAA2c*, *CmIAA16*, and *CmIAA15*), the remaining CmAux/IAA proteins exhibited instability indices exceeding 40, which could classify them as unstable ones. Meanwhile, nuclear localization for most CmAux/IAA proteins could be indicated by the subcellular localization predictions, and *CmIAA2c* and *CmIAA31b* exhibited both nuclear and cytoplasmic distribution. Furthermore, a phylogenetic tree was constructed with the application of 83 Aux/IAA protein sequences from *C. mollissima*, *A. thaliana*, and *O. sativa* (Figure 1; Table S2), which aimed at the exploration of the evolutionary relationships. The analysis revealed four distinct groups (Group I-IV) containing 21, 22, 16, and 24 members, respectively (Figure 1A,C). The proportion of *CmAux*/*IAA* genes in each group was 22.10%, 29.54%, 17.41%, and 30.95%, respectively (Figure 1B).

### 3.2. Gene Structure and Conserved Motif Analysis

**Figure 2:**
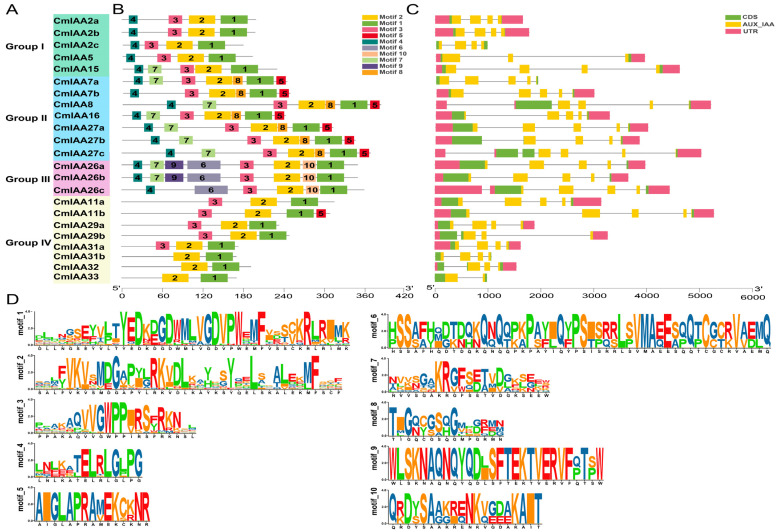
Gene structure and conserved motifs of the Aux/IAA gene family in *C. mollissima*. (**A**) Four subfamily members of the CmAux/IAA gene family. (**B**) Distribution of conserved motifs in CmAux/IAA proteins. (**C**) Gene structure and conserved domains of *CmAux*/*IAAs*. (**D**) The amino acid sequence of ten motifs.

The conserved motifs and gene structures of *CmAux*/*IAA* genes were further analyzed to investigate the structural characteristics of the CmAux/IAA family members (Figure 2). According to the results, members from the same subfamily displayed similar gene structures and conserved motif arrangements. For example, Group I, Group II, and Group III contained common motifs 1, 2, 3, and 4, in which motif 4 had a classical “LxLxLx” motif, which was used for the identification of domain I. Additionally, motif 1 and motif 3 had the classical “GDVP” and “GWPPV”, respectively, which corresponded to domains IV and II, while motif 2 corresponded to domain III. The motif composition analysis revealed distinct patterns among the subgroups: Group I contained all four conserved motifs 1, 2, 3, 4; Group II additionally contained motifs 5, 7, and 8; Group III was characterized by extra motifs 6, 7, 9, and 10; and Group IV predominantly maintained motifs 1,2,3, with the exceptions of *CmIAA33*, *CmIAA32*, and *CmIAA31b* which retained only motifs 1 and 2. Notably, all CmAux/IAA members universally maintained motifs 1 and 2 (Figure 2A,B,D), which could strongly indicate the crucial functions of these motifs during the mediation of specific *ARF* binding interactions. Furthermore, the exon–intron organization of *CmAux*/*IAA* genes was analyzed to characterize their genomic structures (Figure 2C), and it was revealed that all *CmAux*/*IAA* genes contained two to seven exons and one to six introns, four genes in the five members of Group I contained four exons and three introns, five genes in seven members of Group II contained five exons and four introns, two of the three members of Group III contained five exons and four introns, and five of the eight members of Group IV contained four exons and three introns.

### 3.3. Chromosome Location and Collinear Analyses

**Figure 3:**
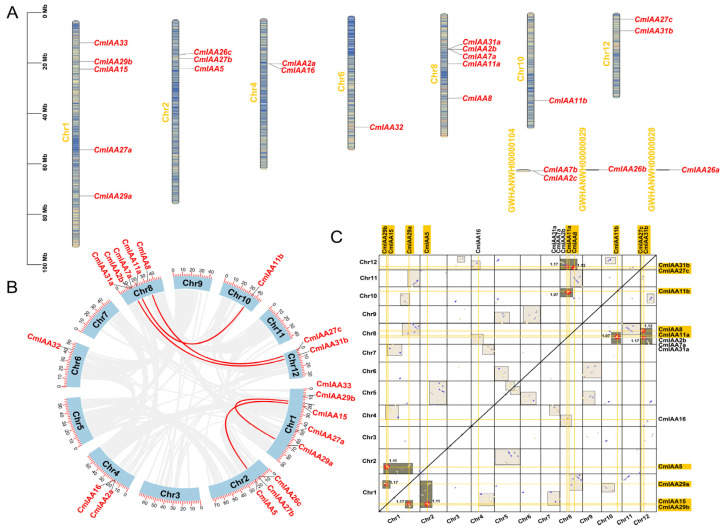
Chromosome distribution and duplication type analysis of *CmAux*/*IAA* genes. (**A**) Chromosomal localization of *CmAux*/*IAA* genes in the *C. mollissima* genome. (**B**) Intra-species collinear relationships among *CmAux*/*IAA* genes. Red lines indicate duplicated *Aux*/*IAA* gene pairs. The number on the circle represents the chromosome number. (**C**) Dot-plot visualization of homologous collinear gene pairs containing CmAux/IAA members. Collinear gene blocks associated with WGD events are highlighted in gray boxes, with median Ks values indicated for each block. Genes potentially originating from WGD events are marked in yellow. The peripheral annotations on the dot-plot exclusively display *CmAux*/*IAA* genes identified through MCScanX analysis as either WGD-derived or segmentally duplicated candidates.

According to the analyses, 19 *CmAux*/*IAA* genes were distributed across seven chromosomes, while *CmIAA2c*, *CmIAA7b*, *CmIAA26a*, and *CmIAA26b* were located on three unanchored scaffolds (Figure 3A). Chromosomes 1 and 8 exhibited the highest number of *CmAux*/*IAA* genes with five CmAux/IAA members, followed by chromosomes 2, 4, and 12 with 3, 2, and 2 *CmAux*/*IAA* genes, respectively, with only one CmAux/IAA member in chromosomes 6 and 10. Notably, gene duplication has been well documented as a key mechanism that could drive the gene family formation, expansion, and functional diversification [45]. Therefore, intra-genomic collinear analysis of the *C. mollissima* genome was performed to clarify the duplication patterns of *CmAux*/*IAA* genes, and five pairs of homologous *CmAux*/*IAA* genes were identified (Figure 3B). Additionally, we further distinguished between WGD and segmental duplication events based on the analysis of both collinear patterns of collinear blocks and median Ks values of homologous gene pairs (Figure 3C), which was based on the methodology of our previous study [46]. It was confirmed that 10 CmAux/IAA members were derived from WGD, seven genes from dispersed duplication, and six genes from segmental duplication, which emphasized the crucial contributions of WGD in the expansion of the CmAux/IAA gene family. In addition, since their non-synonymous (Ka)/substitution sites (Ks) values were below 1 (Table S5), it could be revealed that they were mainly affected by the purification selection during evolution.

**Figure 4:**
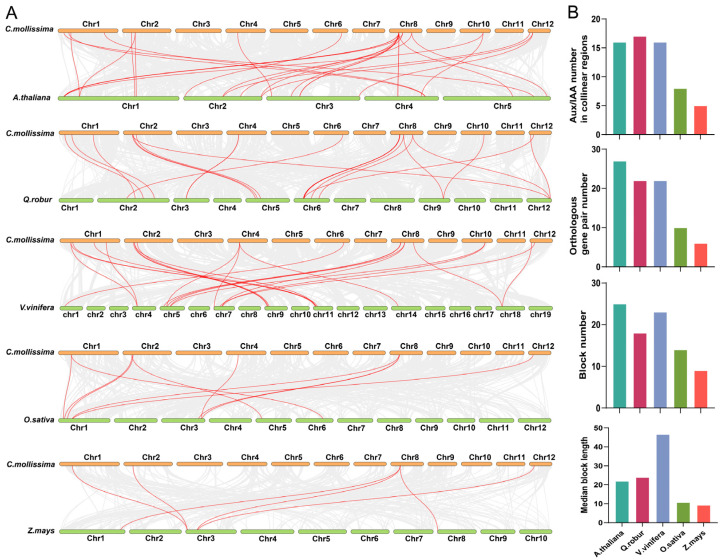
Collinear analyses between *C. mollissima* and five representative plant species (*A. thaliana*, *Q. robur*, *V. vinifera*, *O. sativa*, and *Z. mays*). (**A**) The dual collinear plot between *C. mollissima* and five representative plant species. Gray lines in the background indicate collinear blocks within *C. mollissima* and other plant genomes, while red lines highlight collinear *Aux*/*IAA* gene pairs. (**B**) The *Aux*/*IAA* gene number in collinear regions, orthologous gene pairs number, blocks number, and median block lengths between *C. mollissima* and the other five plant genomes.

*C. mollissima* and five representative plants (*A. thaliana*, *Q. robur*, *V. vinifera*, *O. sativa*, and *Z. mays*) were deeply explored to analyze the evolution of the CmAux/IAA gene family (Figure 4A). There were 16, 17, 16, 8, and 5 *CmAux*/*IAA* genes in the collinear regions of the genomes of *C. mollissima* and the above five plants, respectively (Figure 4B; Tables S6–S10). In addition, there were 27, 22, 22, 10, and 6 orthologous gene pairs between *C. mollissima* and the genomes of five plants, respectively (Figure 4B; Tables S11–S15). These results suggested that the Aux/IAA gene family maintained a better collinear relationship among dicotyledonous plants. Notably, some *CmAux*/*IAA* genes could form multiple collinear gene pairs with other plants, such as those associated with *CmIAA2b*, *CmIAA11a*, *CmIAA7a*, *CmIAA31a*, and *CmIAA31b* in the collinear blocks of *A. thaliana*, and *CmIAA26c* in the collinear block of *O. sativa*. According to the gene balance hypothesis, these genes were confirmed to exhibit crucial functions in the evolutionary expansion of the CmAux/IAA gene family [47]. Notably, certain collinear gene pairs were exclusively conserved between *C. mollissima* and the dicotyledonous plants (*A. thaliana*, *V. vinifera*, *Q. robur*), while they were absent in comparisons with the monocotyledons (*O. sativa* and *Z. mays*). It could be suggested by this pattern that the Aux/IAA gene family might have undergone lineage-specific duplication events during the evolution. Furthermore, the number of collinear blocks containing CmAux/IAA between *C. mollissima* and each comparison genome was 25, 18, 23, 14, and 9, respectively, and the median block lengths were 21.92, 23.944, 46.565, 10.71, and 9.22, respectively (Tables S11–S15). Combined with the previous reports [48,49], these results might be connected with the evolutionary relationships between these plants and the occurrence of WGD events.

### 3.4. Cis-Acting Elements Analysis

**Figure 5:**
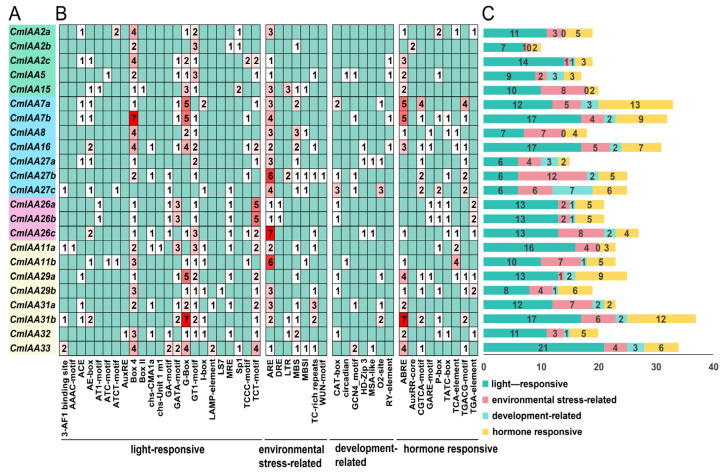
Prediction of *cis*-acting elements in the promoters of *Aux*/*IAA* genes in *C. mollissima*. (**A**) The same subfamily is grouped into the same color. (**B**) The number of different *cis*-acting elements in the promoters of 23 *CmAux*/*IAA* genes is indicated in the chart. The different colors represent the number of *cis*-acting elements. (**C**) The number of various *cis*-acting elements in the promoters of each *CmAux*/*IAA* gene.

Gene expression is regulated by promoter elements, and the analysis of *cis*-acting elements in promoter regions could provide insights into the deep exploration of potential gene functions [50]. In this research, 2000 bp upstream sequences of *CmAux*/*IAA* genes were analyzed to identify putative *cis*-regulatory elements, and 537 *cis*-acting elements were identified across all 23 *CmAux*/*IAA* promoters, which could be classified into four categories (Figure 5A,B; Table S16): Light-responsive elements (23), Development-related elements (7), Stress-responsive elements (7), Hormone-responsive elements (9). Notably, the development-related elements were found to be predominantly enriched in CAT-box, GCN4_motif, and 02-site. In addition, the main enrichment of hormone-responsive elements was ABRE, CGTCA-motif, P-box, TCA-element, and TGA-element, which could regulate abscisic acid (ABA), methyl jasmonate (MeJA), gibberellin (GA), salicylic acid (SA), and auxin (IAA), respectively. Among the 23 *CmAux*/*IAA* genes, eight of them contained auxin-related *cis*-elements (AuxRR-core and TGA-element), with *CmIAA2b*, *CmIAA29b*, *CmIAA26a*, and *CmIAA26b* showing particularly strong auxin responsiveness—each harboring two auxin-related elements in their promoter regions. Furthermore, it could be suggested by the *cis*-acting elements profiling that the CmAux/IAA gene family likely exhibited crucial functions in *C. mollissima* hormone regulation and response to light signaling.

### 3.5. TFs Regulatory Network Analysis, GO/KEGG Enrichment, and PPI Network Analysis

**Figure 6:**
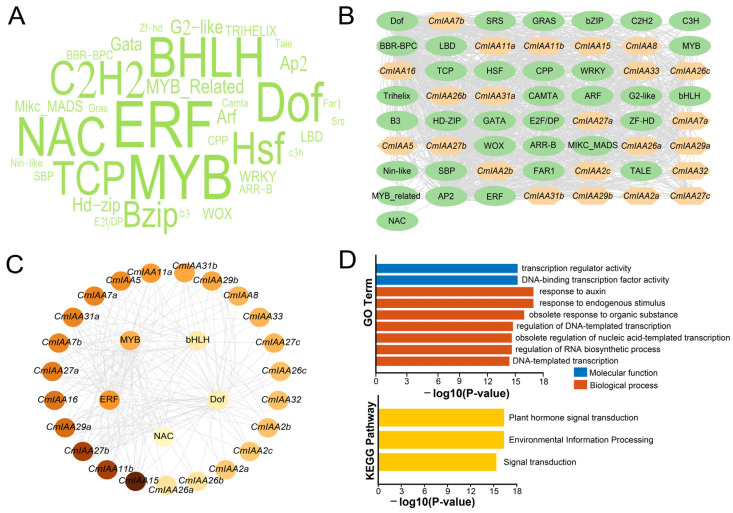
TFs regulatory network analysis and GO/KEGG enrichment of *CmAux*/*IAAs*. (**A**) Wordcloud of predicted TFs interacting with *CmAux*/*IAA* genes. The font size is positively correlated with the number of corresponding TFs. (**B**) The putative TFs regulatory network analysis of *CmAux*/*IAAs*. (**C**) The top five highly enriched and targeted *CmAux*/*IAAs* are shown; the darker the color, the more highly enriched. (**D**) GO/KEGG function enrichment analysis of *CmAux*/*IAAs*.

To further explore the functions of *CmAux*/*IAA*, transcription factors that interacted upstream of *CmAux*/*IAA* genes were predicted (Figure 6A,B; Table S17). According to the results, it was indicated that 167 transcription factors were predicted for the 23 *CmAux*/*IAA* genes, which belonged to 34 different transcription factor families. Most of these transcription factors belonged to ERF (25), followed by MYB (21), bHLH (12), Dof (11), NAC (10), C2H2 (9), TCP (9), HSF (7), and bZIP (6), and the least were found in TALE, GRAS, C3H, B3, Nin-like, FAR1, SRS, CAMTA, ZF-HD, and E2F/DP (all containing only 1 TF). Based on the prediction results, each of the 23 *CmAux*/*IAA* genes predicted at least 13 TFs, with *CmIAA15* predicting the most TFs (41 TFs), followed by *CmIAA11b* and *CmIAA27b* (36 TFs). Additionally, the top five predicted TFs were identified in terms of abundance in ERF, MYB, bHLH, Dof, and NAC (Figure 6C), which might exhibit crucial functions in the regulation of *CmAux*/*IAA*, and the expression of *CmAux*/*IAA* genes might be synergistically regulated by multiple signaling pathways. Overall, the above results further implied the involvement of these transcription factors in *C. mollissima* development or response to environmental stresses, which was based on the interaction with the promoter region upstream of *CmAux*/*IAA*, in order to regulate its expression.

GO/KEGG enrichment analysis can translate the high-throughput data into an understandable biological language, which constructs a bridge between genomic variation and phenotypic changes, and it is especially indispensable in studies of plant development, metabolism, and stress resistance [51]. Among the protein sequences annotated in the GO database (Figure 6D), CmAux/IAA proteins could be categorized into two main groups (molecular functions and biological processes). In terms of molecular functions, *CmAux*/*IAA* only exhibited connections with transcription regulator activity and DNA-binding transcription factor activity. Regarding the biological processes, *CmAux*/*IAA* functions were mainly enriched in response to auxin, endogenous stimulus and obsolete response to organic substance. Additionally, in the protein sequences annotated in the KEGG database (Figure 6D). *CmAux*/*IAA* was mainly enriched in plant hormone transduction, environmental information processing, and signal transduction. Based on the above findings, it was suggested that *CmAux*/*IAA* might exhibit crucial functions in hormone regulatory networks (involved in plant development, environmental response, or signaling pathway interactions). Subsequently, the interaction network of CmAux/IAA proteins was constructed and analyzed, which aimed at the exploration of the potential interactions (Figure S1; Table S18). According to the results, 15 CmAux/IAA proteins were predicted, among which 12 CmAux/IAA proteins formed complex protein interaction networks among themselves and with proteins (such as ARF, B3, and WRKY). Regarding the CmAux/IAA proteins, *CmIAA27c* was located at the core of this interaction network and could interact with nine proteins. Functionally, this protein interaction network possessed a large number of B3 proteins, alongside a C-terminal Aux/IAA-interacting domain that could promote interactions between ARFs and Aux/IAA inhibitors, which could regulate the growth hormone response element (TGTCTC) selective activation [52]. In addition, CmIAA33 could only interact with GWHTANWH022998 (B3) and GWHTANWH023593 (Aux/IAA). Furthermore, the interaction between CmIAA27a and CmIAA27b was associated with two proteins, which were composed of GWHTANWH004534 (ARF) and GWHTANWH018902 (ARF). Additionally, Aux/IAA proteins could interact with ARF proteins through a homologous structural domain at the C-terminus, thus preventing the direct transcriptional regulation of growth hormone-responsive genes by ARF [53].

### 3.6. Expression Patterns of CmAux/IAA Genes in Different Tissues and Under Abiotic Stress

**Figure 7:**
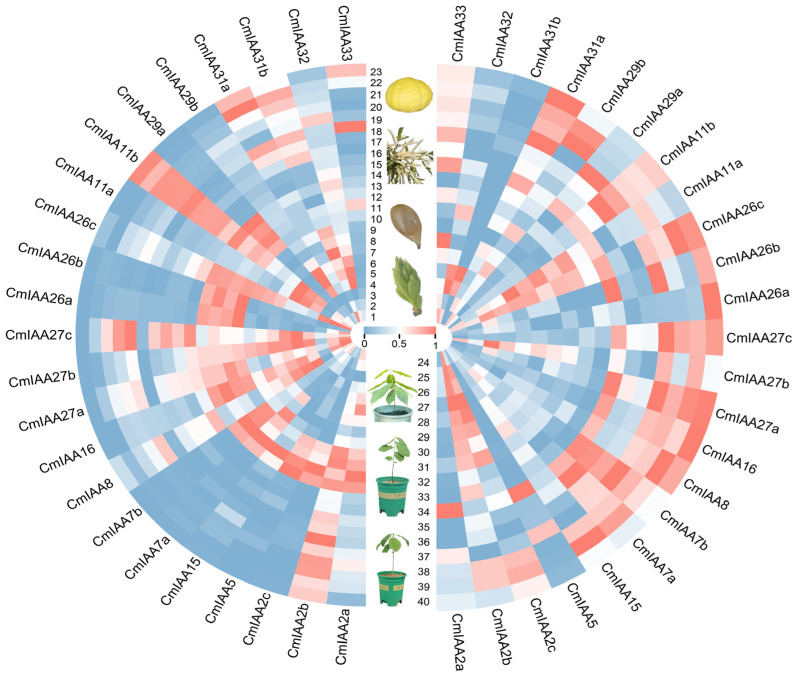
Expression of *CmAux*/*IAA* genes in various tissues and under abiotic stress. 1–3: Gene expression in buds 20, 25, and 30 days after flowering. 4–9: Gene expression in fertile and abortive ovules on 15-July, 20-July, and 25-July. 10–13: Gene expression in first and secondary female flowering, first and secondary male flowering. 14–23: Gene expression in seed kernels of the cultivars ‘Yanshanzaofeng’ and ‘Yanlong’ 60, 70, 80, 90, and 100 days after flowering. 24–33: Gene expression in leaves of cultivars ‘Dabanhong’ and ‘Yanshanzaofeng’ treated with drought for 0, 10, 20, 30, and 40 days. 34–40: Gene expression in leaves of cultivar ‘Yanshanzaofeng’ at room temperature (25 °C), treated with heat stress (45 °C) for 4, 8, and 12 h, and with cold stress (−15 °C) for 5, 10, and 15 h.

Using data from public databases, the expression profiles of *CmAux*/*IAAs* in different tissues were analyzed, including buds (three developmental stages), ovules (fertile and abortive ovules at three developmental stages), flowers (male flowers: first and secondary flowers, female flowers: first and secondary flowers), seed kernels (five developmental stages of two *C. mollissima* cultivars ‘Yanshanzaofeng’ and ‘Yanlong’) (Figure 7). Except for *CmIAA15* and *CmIAA11b*, 21 *CmAux*/*IAAs* exhibited high expression at 20 days of floral bud development followed by continuous down-regulation, suggesting that *CmAux*/*IAAs* are actively involved in the early differentiation of *C. mollissima* buds. The expression of *CmIAA29b* was significantly higher in fertile ovules at 15, 20, and 25 days of development compared to abortive ovules at the corresponding stages, indicating its potential role in ovule fertility. All *CmAux*/*IAAs* genes were expressed in flowers, with minimal differential expression between male and female flowers for the same gene. Furthermore, the expressions of *CmIAA27a*, *CmIAA27b*, and *CmIAA27c* were significantly down-regulated at five stages of seed kernel development. These results demonstrate that *CmAux*/*IAAs* genes may play crucial roles in the growth and development of various tissues of *C. mollissima*.

The expression profiles of *CmAux*/*IAAs* under drought stress (five varying degrees of drought across two varieties, ‘Yanshanzaofeng’ and ‘Dabanhong’), heat stress, and cold stress (four different degrees of cold stress (−15 °C) and heat stress (45 °C), respectively) were analyzed. Except for *CmIAA2b*, *CmIAA2c*, *CmIAA15*, *CmIAA7a*, *CmIAA7b*, and *CmIAA27b*, the expression of other *CmAux*/*IAAs* genes showed no significant differences between the two cultivars under drought treatment. Under heat stress, the expression of most *CmAux*/*IAAs* genes was down-regulated, particularly *CmIAA5* and *CmIAA16*, which exhibited a significant decrease in expression with prolonged treatment duration. Under cold stress, the expression of *CmIAA2a* and *CmIAA5* was significantly down-regulated over time, whereas *CmIAA2b*, *CmIAA2c*, *CmIAA15*, and *CmIAA29b* were transiently up-regulated at 5 h and 10 h before being down-regulated at 15 h. These results suggest that *CmAux*/*IAAs* may play important roles in plant responses to abiotic stress.

### 3.7. Phenotypic Changes During the Development

**Figure 8:**
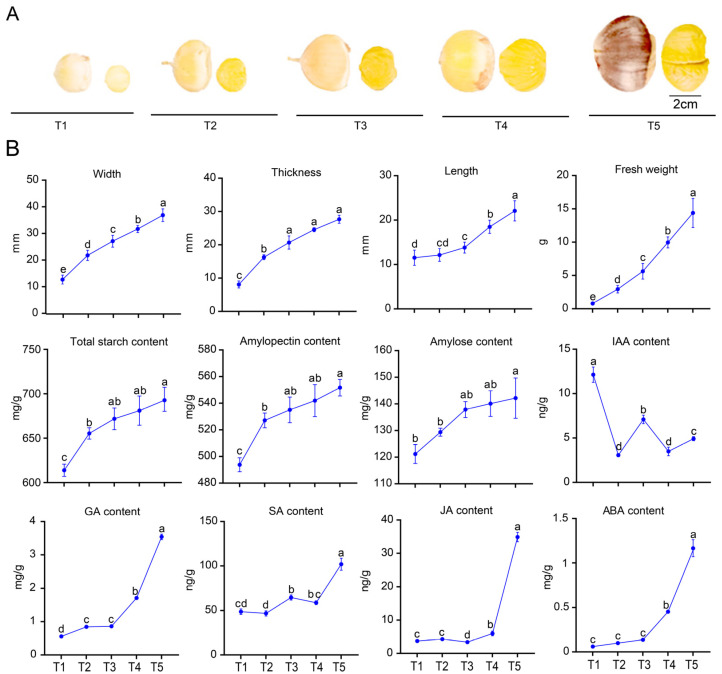
The changes in morphology and physiological indicators of *C. mollissima* nuts and seed kernels at different developmental stages (T1–T5 represent 60, 70, 80, 90, and 100 days after flowering, respectively). (**A**) Morphological images of nuts and seed kernels of ‘Liuyuebao’ *C. mollissima* at T1–T5 developmental stages. (**B**) The morphological indexes (width, thickness, length, fresh weight) and physiological indexes (total starch, amylopectin, amylose, IAA, GA, SA, JA, and ABA content) of seed kernels in the T1–T5 stages of *C. mollissima*. The small letters indicate the significance of differences between physiological indicators in five stages.

To analyze the development characteristics, the seed kernels were monitored in terms of morphological size, starch content, and growth hormone in five stages after the flowering of *C. mollissima* ‘Liuyuebao’ (Figure 8A,B). According to the results, the overall trend of *C. mollissima* seed kernels in terms of morphological size, starch, and hormone content exhibited an increasing trend with the change of development time, and it reached the maximum value at stage T5. For instance, the average fresh weight of the seed kernels increased from 0.78 g at stage T1 to 14.39 g at stage T5; the total starch content of the seed kernels increased from 614.96 mg/g at stage T1 to 693.85 mg/g at stage T5, and the amylopectin content was 3–4 times higher than the amylose content; the seed kernels GA content level increased from 0.56 mg/g at stage T1 to 3.54 mg/g at stage T5. However, the overall trend of IAA hormone content of the seed kernels exhibited a decreasing trend from the highest level at stage T1 (12.13 ng/g) to the lowest at stage T2 (3.06 ng/g). In addition, changes in the seed kernel size (length, width, thickness, and fresh weight) exhibited the most significant increasing rate between T1 and T2, which could suggest more biological activity of the seed kernels during this stage. Furthermore, little variation could be found in length during the pre-developmental stage, and the fastest growth rate could be found between stages T3 and T4. In hormone levels (GA, SA, JA, ABA), the fastest growth rate was observed between stages T4 and T5, which could be attributed to the promotion of the seed kernel maturation by hormones. In addition, the changes in IAA hormone content exhibited a first rise and then a decreasing trend, and its rapid decline could be found between stages T1 and T2. The content of starch (total starch, amylopectin, and amylose) exhibited the fastest growth rate during stages T1 and T2, which could indicate the rapid starch accumulation during this stage.

### 3.8. RNA Sequencing and DEGs Identification

**Figure 9:**
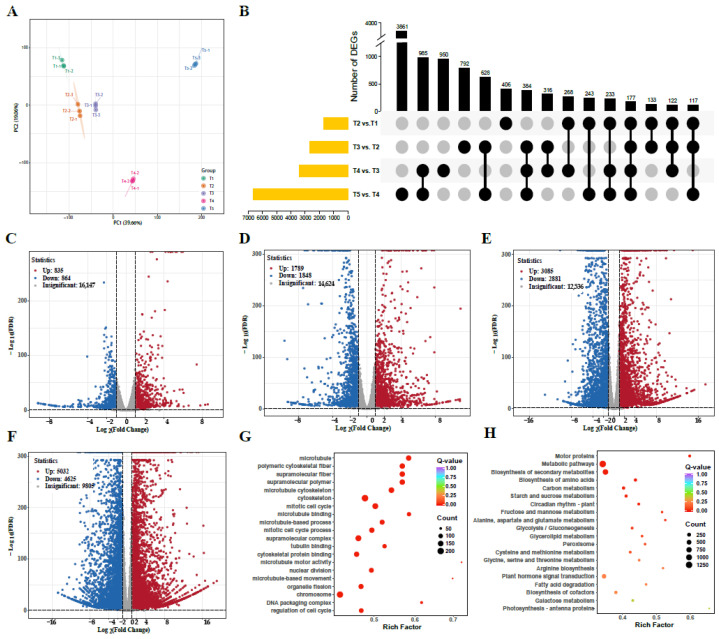
Transcriptome analysis results. (**A**) PCA analysis of transcriptome data. (**B**) UpsetR analysis of DEGs at different developmental stages. (**C**) DEGs between stage T1 and T2. (**D**) DEGs between stage T1 and T3. (**E**) DEGs between stage T1 and T4. (**F**) DEGs between stages T1 and T5. (**G**) GO enrichment of DEGs at stage T1 vs. T5. (**H**) KEGG enrichment of DEGs at stage T1 vs. T5.

For the analysis of the potential impact of genes during the development, mRNA was extracted from *C. mollissima* seed kernels from five developmental stages, and 15 cDNA libraries were constructed (three replicates per treatment). Transcriptome sequencing produced high-quality clean reads (totaling 161.31 Gb) with base quality of Q20 ≥ 97.61%, Q30 ≥ 92.95%, and GC content ranging from 43.57% to 45.43% (Table S19). According to the results, the comparisons with the reference genome ranged from 88.51% to 89.74% (Table S20). Notably, PCA analysis demonstrated significant differences in gene expression across the five stages (Figure 9A). To investigate the dynamic changes in DEGs during *C. mollissima* seed kernel development, we identified DEGs between adjacent developmental stages (Figure 9B). Notably, our comparative analysis between stage T1 and the subsequent four stages (T2–T5) revealed 912 significant DEGs (Figure S2). In addition, significant changes could be observed in gene expression during the development: 835, 1789, 3085, and 5032 genes were up-regulated, while 864, 1848, 2881, and 4625 genes were down-regulated in the T1 vs. T2, T1 vs. T3, T1 vs. T4, and T1 vs. T5 stages, respectively (Figure 9C–F). According to these results, it was demonstrated that the down-regulated DEGs outnumbered up-regulated DEGs during the T1 vs.T2 and T1 vs. T3 developmental phases, whereas the opposite pattern emerged in the T1 vs. T4 and T1 vs. T5 stages, and the up-regulated DEGs surpassed the down-regulated ones. Furthermore, it could be revealed by GO and KEGG enrichment analyses of T1 vs. T5 (Figure 9G,H) that DEGs were mainly enriched in microtubule, supramolecular polymer, supramolecular fiber, polymeric cytoskeletal fiber, microtubule cytoskeleton, cytoskeleton, Metabolic pathways, and Biosynthesis of secondary metabolites.

### 3.9. Expression Patterns of CmAux/IAAs and Their Connections with Phenotypic Indicators

**Figure 10:**
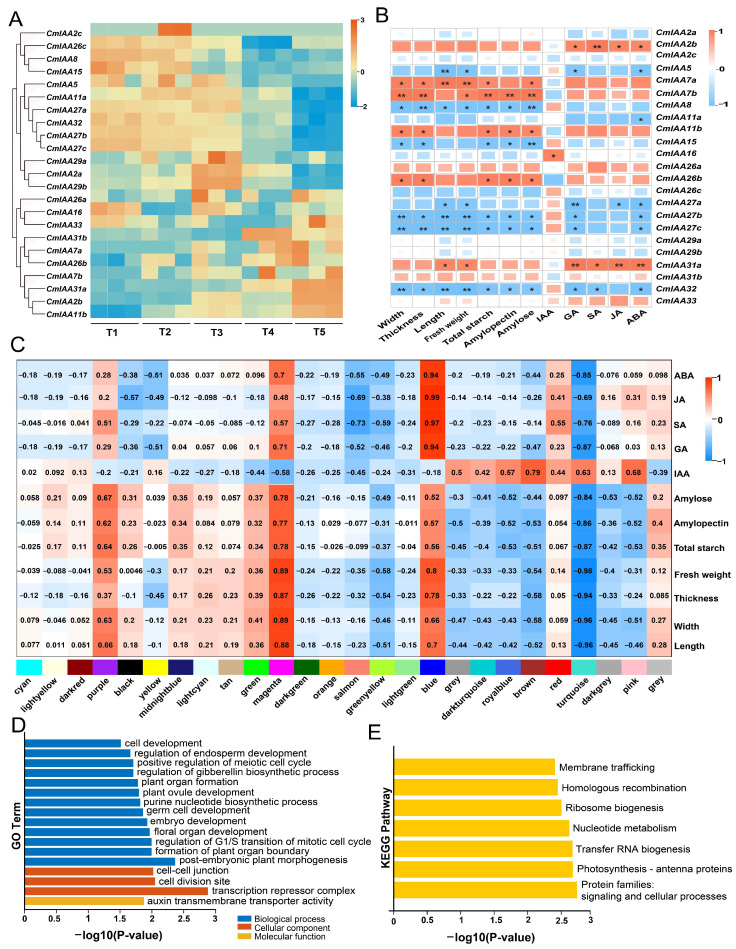
Expression patterns of CmAux/IAAs and their association with growth indexes, and functional enrichment of turquoise module genes in WGCNA analysis. (**A**) The expression profiles of CmAux/IAAs at five stages of seed kernel development of *C. mollissima*. (**B**) The correlation between growth indicators and *CmAux*/*IAA* expression levels at five stages of seed kernel development of *C. mollissima*. ** and * were used to mark genes and physiological indicators that have extremely significant and significant correlations, respectively. (**C**) The correlation of the identified modules with the growth indicators at five stages of seed kernel development of *C. mollissima*. (**D**) GO enrichment analysis of the genes in turquoise modules. (**E**) KEGG enrichment analysis of the genes in turquoise modules.

The involvement of *Aux*/*IAA* genes in fruit development and ripening has been mentioned in numerous studies [54,55]. Notably, the expression of the Aux/IAA gene family exhibited a significantly varying trend during the five stages of development (Figure 10A). For example, the expression of *CmIAA31a*, *CmIAA2b*, *CmIAA7a*, *CmIAA7b*, and *CmIAA11b* continued to be up-regulated with the seed kernel development, and the FPKM values of *CmIAA31a* were significantly varied among the five stages (values of 0.48, 0.58, 1.21, 6.29, and 25.32). Especially, the FPKM values at stage T5 were about 52 times that at stage T1. Notably, the FPKM values of *CmIAA7a* and *CmIAA7b* were 0 at stage T1, which exhibited little variation during the next four developmental stages. In contrast, the expression of *CmIAA27b*, *CmIAA27c*, *CmIAA32*, and *CmIAA8* continued to be down-regulated, especially for *CmIAA27b*, which exhibited an FPKM value of about 35 times at stage T1 as compared to stage T5. In addition, some genes showed dynamic changes in expression during the five developmental stages. For instance, the FPKM of *CmIAA27a* was down-regulated and then up-regulated, followed by down-regulation again, while the FPKM of *CmIAA5* was up-regulated at stage T2 and then decreased to 0 at stage T5.

The underlying connections between *CmAux*/*IAA* expression levels and physiological indicators (seed kernel size, starch, and hormone contents) were explored (Figure 10B) to clarify the functions of *CmAux*/*IAA* genes. Notably, the *CmAux*/*IAA* genes exhibited a strong connection with the fruit size, especially fresh weight, and these metrics were relevant to the evaluation of fruit development [54,56,57]. Based on these findings, it was indicated that *CmIAA7a* exhibited a highly significant positive connection with the seed kernels’ fresh weight, alongside the significant positive correlation between *CmIAA7b* and *CmIAA31a* with the seed kernels’ fresh weight. However, *CmIAA27b*, *CmIAA27c*, and *CmIAA32* exhibited a highly significant negative connection with the fresh weight, while *CmIAA27a*, *CmIAA5*, and *CmIAA8* exhibited a significant negative connection with the fresh weight. In addition, based on the combination of WGCNA analysis with RNA-seq data from the five stages, it could be determined that *CmIAA27b* and *CmIAA27c* were part of the turquoise module, and this module exhibited a negative connection with the seed kernels’ fresh weight and starch content (Figure 10C; Figure S3). In the GO/KEGG enrichment analysis of the turquoise module, it was found that the turquoise module was mainly associated with the transcription repressor complex, cell division, plant organ, and auxin transmembrane transporter activity (Figure 10D,E).

### 3.10. Expression Analysis of CmAux/IAA Genes During Seed Kernel Development by RT-qPCR

**Figure 11:**
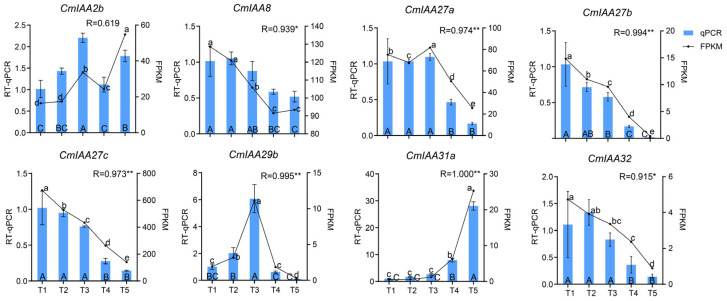
RT-qPCR analysis of *CmAux*/*IAA* genes at T1-T5 stages of kernel development in *C. mollissima* seeds. The small letters indicate the significance of differences between FPKM of transcriptome data from five stages, while uppercase letters indicate the significance of differences between RT-qPCR results from five stages. ** and * were used to mark the expression levels of RT-qPCR and RNA-seq that have extremely significant and significant correlations, respectively.

RT-qPCR experiments were performed on the *CmAux*/*IAA* genes at five stages of *C. mollissima* seed kernel development to verify the accuracy of the RNA-seq data (Figure 11). Except for *CmIAA2b*, the RT-qPCR results of the other seven genes exhibited significant positive connections with the RNA-seq sequencing data, which could emphasize the accuracy of the RNA-seq analysis. For instance, the expression of *CmIAA2b* was first down-regulated and then up-regulated after reaching a maximum at stage T3, while the *CmIAA31a* was continuously up-regulated and reached a peak at stage T5. Conversely, the expression of *CmIAA27b* and *CmIAA27c* was continuously down-regulated. Additionally, the expression of *CmIAA27a* and *CmIAA29b* was first up-regulated and reached the maximum at stage T3, followed by a down-regulated trend. The expression of *CmIAA32* and *CmIAA8* reached the maximum at stage T2 and then continued to be down-regulated. Overall, the RT-qPCR assay could also demonstrate the expression of *CmIAA27a*, *CmIAA27b*, and *CmIAA27c* in seed kernel development.

## 4. Discussion

Auxin could regulate multiple aspects of fruit development, including fruit set, growth, maturation, and abscission, and the crucial functions of *Aux*/*IAA* genes have been well documented [2,19,58]. However, the specific functions of *Aux*/*IAA* mediated by auxin signaling remain unexplored in *C. mollissima*. A systematic characterization of the CmAux/IAA gene family in *C. mollissima* was conducted in this research, which could provide insights into their underlying functions. Based on the findings, a fundamental framework was established for future investigations into the functional significance of *Aux*/*IAA* genes.

### 4.1. The Molecular Characteristics of Aux/IAA Genes

*C. mollissima* possessed 23 members of the Aux/IAA gene family, with one Aux/IAA conserved domain in each of them. Comparatively, the proteins encoded by the 23 genes exhibited significant differences in the aspects of amino acids number and molecular weight, with the vast majority of the proteins holding an isoelectric point of greater than 7, an instability index of greater than 40, and a total mean value of the hydrophilicity of the proteins below 0 (Table S1). These results collectively demonstrated that Aux/IAA proteins were unstable, hydrophilic, and alkaline in nature. Furthermore, the subcellular localization of Aux/IAA proteins was in the nucleus of major cells, which could regulate gene expression and participate in certain activities (such as cellular metabolism and genetics) [59]. CmAux/IAA members divided into four subfamilies, which formed a phylogenetic tree with *A. thaliana* and *O. sativa* (Figure 1). Specifically, members in the same subfamily exhibited similar gene structures and conserved motifs, and the four groups of members are highly conserved. Group I contained all four conserved motifs 1, 2, 3, and 4; Group II additionally contained motifs 5, 7, and 8; and Group III was characterized by extra motifs 6, 7, 9, and 10. These three subfamilies collectively comprised 15 CmAux/IAA members, all of which shared the core motifs 1, 2, 3, and 4, corresponding to domains IV, III, II, and I, respectively. Therefore, 15 CmAux/IAAs were typical Aux/IAA proteins containing a complete set of four domains, which could exert transcriptional repression and would be rapidly degraded during growth hormone signaling, and they were involved in the growing processes of growth hormone-regulated plants (Figure 2) [7]. In contrast, eight members of Group IV were lacking in either domain I or I and II, and they might be atypical Aux/IAA: five of these genes (*CmIAA29a*, *CmIAA29b*, *CmIAA31a*, *CmIAA11a*, and *CmIAA11b*) lacked domain I, which could indicate the inability of these proteins to attract the TOPLESS and their absence in typical auxin signal transduction. Additionally, three members (*CmIAA32*, *CmIAA33*, and *CmIAA31b*) lacked both domain I and II, and deletion of these structures could prolong the half-life of the proteins versus other classical Aux/IAA proteins, resulting in disruption of auxin physiology and auxin-related aberrant phenotypes [60]. Five gene pairs originated from WGD were identified in the *CmAux*/*IAA* genes based on the collinear analysis, and WGD served as the main driver in gene duplication events (Figure 3B,C; Table S5). Based on the presence of many *cis*-elements related to hormone regulation in the promoter regions of CmAux/IAAs (Figure 5; Table S16), including ABRE (ABA), P-box, TATC-box (GA), TCA-element (SA), and TGA-element (IAA), it could be suggested that they might be involved in hormonal responses. In addition, an interaction network was constructed between *CmAux*/*IAA* genes and TFs, which might affect their promoter regions (Figure 6A–C; Table S17). Furthermore, the top 5 TFs in abundance (ERF, MYB, bHLH, Dof, and NAC) exhibited crucial functions in plant development, metabolism, and stress response through the regulation of gene networks [61,62,63,64], which could indicate the active involvement of *CmAux*/*IAA* genes in plant development (Figure 6B–D). Given the importance of protein interactions in the study of many physiological processes (Figure S1; Table S18) (such as signal transduction and gene expression regulation) [65], it was revealed in protein interaction network analysis that *CmIAA27a*, *CmIAA27b*, and *CmIAA27c* might participate in regulating of the growth hormone signaling pathway mainly based on the ARF protein family.

### 4.2. Analysis of the Evolution and Expansion of the CmAux/IAA Gene Family

With the recent advancements in whole-genome sequencing technologies, the identification of Aux/IAA gene families in individual plant species has become a crucial approach to explore their functional roles in plants [66]. The number of identified Aux/IAA members in *C. mollissima* (23) is relatively lower compared to other species: *A. thaliana* (29) [16], *O. sativa* (31) [17], *S. lycopersicum* (26) [18], *M. pumila* (33) [19], *Z. mays* (40) [20]. This reduction may result from gene loss following WGD events in the Fagaceae lineage [67]. Comparative analysis of exon–intron structures across plant species revealed significant variation in gene architecture [16,17,18,19,20]. Among 23 CmAux/IAA members examined, nine genes exhibited the conserved five-exon/four-intron organization—a pattern consistently observed in *Aux*/*IAA* genes from both dicotyledons (*A. thaliana*) and monocotyledons (*O. sativa*) [16,17]. This structural conservation suggests their derivation from a common ancestral gene, with the archetypal configuration likely predating the dicotyledons and monocotyledons divergence [17,68]. Seven *CmAux*/*IAA* genes show either exon duplications or losses compared with their orthologous genes in *A. thaliana*. Notably, five *CmAux*/*IAAs* displayed exon gain events, potentially reflecting adaptive evolution to meet the unique developmental demands of woody plants, including perennial growth cycles and secondary growth regulation [69,70]. In contrast, *CmIAA32* and *CmIAA33* displayed reduced exon numbers, likely resulting from transposable element insertions disrupting splice sites [71]. Interestingly, these two genes retain only domains III and IV, suggesting potential functional specialization through structural simplification [72].

Thylogenetic analysis results indicated that *CmAux*/*IAA* and *AtAux*/*IAA* genes are almost all located in very close branches, suggesting that they have closer genetic examples compared to *Aux*/*IAA* genes in *O. sativa* (Figure 1). The highly similar sequences and gene structures could imply the performance of similar functions [73]. Notably, the responsiveness of the *IAA2* promoter to IAA and IBA in the Group I branch was differentially affected in *A. thaliana* roots and shoots by flavonoids [74]. *CmIAA2s* (*CmIAA2a*, *CmIAA2b*, *CmIAA2c*) were located in the same branch as *IAA2*, and these two might exhibit similar functions. Within Group II, *IAA8* might be affected by the JA level through its interaction with ARF6/8 proteins, which exhibited certain functions in floral organ development through changes in JA levels [75]. Additionally, *CLSY1* could mediate transcriptional repression of *IAA27*, thus promoting lateral root development in *A. thaliana* under potassium-deficient conditions, in order to maintain growth under low-potassium stress conditions [76]. Based on the above findings, *CmIAA8* and *CmIAA27s* (*CmIAA27a*, *CmIAA27b*, *CmIAA27c*) located in the Group II branch might exhibit certain functions in lateral root and floral organ development. In Group III, *IAA26* was a putative regulator of auxin response genes, which was involved in the development of plants [77], and *CmIAA26s* (*CmIAA26a*, *CmIAA26b*, *CmIAA26c*) in Group III were involved in the development of plants. Regarding Group IV, *IAA29* could promote stem cell death in *A. thaliana* roots under DNA stress [78]. Furthermore, expression of *IAA31* may be specifically enhanced in seeds and siliques in *A. thaliana* [79]. Notably, it was newly discovered that auxin signaling mechanisms could stabilize non-classical *IAA32* and *IAA34* transcriptional repressor proteins to regulate gene expression, ultimately inhibiting the growth [9]. Therefore, the functions exerted by *CmIAA29s* (*CmIAA29a*, *CmIAA29b*), *CmIAA31s* (*CmIAA31a*, *CmIAA31b*), *CmIAA32*, and *CmIAA34*, which were located in the Group IV branch, might be influenced by the auxin signaling pathway. Subsequently, the evolutionary trajectory of *Aux*/*IAA* genes was investigated by analyzing the collinear connections between *C. mollissima* and other species, including three dicotyledons (*Q. robur*, *A. thaliana*, *V. vinifera*) and two monocotyledons (*O. sativa* and *Z. mays*) (Figure 4; Tables S6–S10). It was revealed that the collinear connections between dicotyledons were more robustly preserved. Notably, the collinear regions of *C. mollissima* with *Q. robur* (17), *A. thaliana* (16), and *V. vinifera* (16) contained higher counts of *CmAux*/*IAA* genes, and some of the collinear gene pairs (existed with dicotyledonous plants) were absent between *C. mollissima* and monocotyledonous plants, which could suggest that the predominant occurrence of Aux/IAA gene family’s duplications was in dicotyledons (Figure 4). Several *CmAux*/*IAA* genes (such as *CmIAA2b*) could form multiple collinear gene pairs with genes from other plants (e.g., *Q. robur* and *A. thaliana*), which suggested they maintained a higher retention in these plants. This retention is based on the gene balance hypothesis, underscoring their crucial functions in the evolution [47].

Gene duplication is a key mechanism in gene family expansion, which provides necessary genetic materials for the emergence of new genes and functional diversification [80]. In this research, the duplication patterns of *CmAux*/*IAA* genes were analyzed to clarify the driving forces during the expansion. In *C. mollissima*, 23 *Aux*/*IAA* genes originated from WGD or segmental (16) and dispersed duplication (7) (Figure 3B,C). Ten *CmAux*/*IAAs* originated from WGD were identified, according to the complementarity of the collinear blocks formed by WGD and Ks values. Therefore, it was believed that WGD could serve as the main driver of the expansion of the CmAux/IAA gene family. Commonly, WGD is known to affect the genome structure by doubling the number of genes and the genome size. Meanwhile, gene redundancy could remove the selective constraint and provide more genetic resources, which could enable faster genetic innovation [81,82]. Therefore, WGD might result in genomic assemblies that generate evolutionary novelty, and it might serve as a catalyst for functional diversification. Notably, the application of segmental duplications as a complex network could provide more possibilities for future research. For instance, the growth and evolution of a network could be analyzed for any well-annotated genome [83]. Furthermore, dispersed duplication is prevalent in different plant genomes, and the dispersed duplicates make up a substantial part of the duplicated gene population [84].

### 4.3. Expression Pattern of CmAux/IAA Genes During Seed Kernel Development

According to the relevant research, many Aux/IAA gene families have been identified to participate in fruit development and ripening [15,58,73]. For instance, it was revealed that some Aux/IAA participated in regulating fruit size [54,85]. However, the specific functions of *Aux*/*IAA* genes remained unclear in *C. mollissima*. Therefore, the seed kernel shape indices, starch content, phytohormone levels, and CmAux/IAA transcriptional profiles across five developmental stages were deeply investigated in this research (Figure 10). Specifically, seed kernel size serves as a key indicator of yield and quality in angiosperms, and the final size of the fruit was mainly dependent on the coordinated control of cell division and cell expansion [86]. Additionally, several factors exhibited effects on the seed kernel. For instance, starch was a major component and exhibited a rapid accumulation rate in the development of the seed endosperm. Specifically, hormones exhibited a strong connection with the control of the different stages of seed kernel growth, and the degree of expression of Aux/IAA family genes would be affected by different hormones, which underscored the importance of the analysis regarding its accumulation pattern during the reproductive stages [56,87]. Notably, *CmIAA31a* exhibited a significant positive connection with the shape index, starch, and hormone content (Figure 10B), alongside a highly significant positive connection with GA, JA, and ABA, which could indicate the crucial functions of *CmIAA31a* in the regulation of the development and metabolism. In contrast, *CmIAA27b* and *CmIAA27c* exhibited a highly significant negative connection with the fresh weight, and their expression was consistently down-regulated throughout the development, which was consistent with the expression of *VcIAA27* during *Vaccinium uliginosum* fruit development (Figure 10B) [73]. In other crops, some studies focused on the *IAA27* gene functions in fruit development. For instance, the *SIIAA27* gene was highly expressed in ripening fruits, and the down-regulation of *SIIAA27* resulted in smaller *S. lycopersicum* fruits [88], while two *VvIAA27s* (*Vv11s0016g03540* and *Vv14s0030g00110*) in *V. vinifera* expression exhibited a significant increasing trend [89]. In this research, *CmIAA27b* and *CmIAA27c* were down-regulated, alongside an increase in the seed kernel size. Based on sequence homology and phylogenetic analysis, alongside the gene structural characterization, it was concluded that *CmIAA27b* and *CmIAA27c* exhibited similar functions to *VcIAA27*, and they showed different functions from *SIIAA27* and *VvIAA27s*, which could suggest the existence and specific functions of *IAA27* in different species. Furthermore, auxin could promote the fruit size enlargement by increasing the number of cell layers in the pericarp and expanding the placenta [90]. Within this research, the down-regulation of *CmAux*/*IAAs* expression exhibited a positive connection with the auxin content, alongside a negative connection with the other four hormones (GA, SA, JA, and ABA). This suggested that the fruit size might be compensatorily driven by alternative hormones or metabolic pathways under low IAA conditions, which could facilitate the morphological changes [91]. Additionally, WGCNA analysis revealed that *CmIAA27s* (*CmIAA27a*, *CmIAA27b*, *CmIAA27c*) were in the turquoise module, which exhibited crucial functions in the regulation of cell division, and auxin transmembrane transporter activity was enriched in genes that regulated cell division and auxin (Figure 10C–E). According to the results, the MeJA progenitors (CGTCA-motif, TGACG-motif) related to fruit expansion, alongside GA progenitors (P-box, TATC-box) related to cell division, were found in the promoter region of *CmIAA27s* (Figure 5). It could be suggested that the expression of *CmIAA27s* might be regulated through MeJA and GA signaling pathways. In summary, the down-regulation of *CmIAA27s* expression could be influenced by alternative signaling pathways, which could prevent their high expression from reducing the size of *C. mollissima* seed kernel, and the precise mechanisms required further investigation. Additionally, it was confirmed by RT-qPCR analysis that the expression patterns of *CmAux*/*IAA* genes across the five kernel developmental stages were consistent with the RNA-seq results (Figure 10A and Figure 11), which could provide support for the potential functions of *CmIAA27a*, *CmIAA27b*, and *CmIAA27c*. This study provides insights into the potential mechanisms underlying the dynamic development of Chinese chestnut seed kernel, based on the comprehensive analysis of morphological features, phytohormone profiles and gene expression patterns. More in-depth mechanistic validation, including hormone treatment, gene manipulation (over-expression/CRISPR) and protein interaction analysis, will undoubtedly further strengthen the conclusions. These studies are currently limited by the technical challenges of the Chinese chestnut transformation system and the perennial nature of the plant species requiring long-term experiments. Our subsequent work will center on the validation of candidate genes for heterologous and Chinese chestnut native transformation, as well as protein interactions, which will help to resolve their specific functions in Chinese chestnut seed kernel development at the molecular level.

## 5. Conclusions

Overall, a total of 23 CmAux/IAA gene family members were identified and analyzed in this research. Based on the phylogenetic analysis, the CmAux/IAA gene family was divided into four groups, and the characteristics of each group were summarized. Additionally, the gene structures and conserved motifs of *CmAux*/*IAA* genes were analyzed, alongside a summary of their general features. Subsequently, the functions of the *CmAux*/*IAA* genes were further explored based on the relevant analyses (including collinear, promoter *cis*-acting element, transcription factor, functional enrichment, and protein interaction analyses). Based on the findings, we hypothesized that members of the CmAux/IAA gene family exhibited certain functions in the growth, development, and hormone regulation of *C. mollissima*. Furthermore, transcriptome analysis and RT-qPCR validation showed that 23 members of the CmAux/IAA gene family were differentially expressed in *C. mollissima* seed kernels during five developmental stages, alongside the identification of actual functions of *CmIAA27a*, *CmIAA27b*, and *CmIAA27c* in the regulation of the seed kernel size. However, their specific molecular mechanisms in the mediation of seed kernel development need to be further analyzed. Based on the above findings, this study could provide valuable insights into the further study of Aux/IAA gene family members and be a basic reference for the deep exploration of the functions of Aux/IAA in regulating the development of seed kernels.

## Data Availability

All data generated or analyzed during this study are included in this published article and its supplementary information files. The RNA-seq data generated by this study were uploaded to NCBI and can be accessed through the BioProject login number PRJNA1269733.

## Supplementary Materials

The following supporting information can be downloaded at: https://www.mdpi.com/article/10.3390/biology14070806/s1, Figure S1: CmAuxIAA protein interaction network analysis; Figure S2: Venn diagram of DEGs in T1 stage and the other four (T2–T5) stages. Figure S3: The clustering dendrogram of genes identifying the WGCNA modules; Table S1: Accession numbers and characteristics of 23 *CmAux*/*IAA* genes in *C. mollissima*; Table S2: Aux/IAA protein sequence from *A. thaliana* and *O. sativa;* Table S3: Corresponding accession numbers of transcriptome data obtained from NCBI in this study; Table S4: Primers used for qPCR analysis; Table S5: The duplication model and calculation of Ka and Ks ratios of duplicated *CmAux*/*IAA* gene pairs. Tables S6–S10: The orthologous gene pairs containing CmAux/IAA(16) in the cross of *C. mollissima* and *A. thaliana*, *Q. robur*, *V. vinifera*, *O. sativa*, *Z. mays*; Tables S11–S15: The collinear blocks containing CmAux/IAA identified betweent *C. mollissima* and *A. thaliana*, *Q. robur*, *V. vinifera*, *O. sativa*, *Z. mays*; Table S16: Information of *cis*-acting elements predicted from 2000 bp upstream regions of 23 *CmAux*/*IAA* genes; Table S17: Predictive transcription factor (TFs) analysis of *CmAux*/*IAA* genes; Table S18: CmAux/IAA protein interaction network analysis; Table S19: Summary of transcriptome sequencing; Table S20: Mapping rate range information statistics with reference genome.
