# Supplementary figures and images for: Genome-Wide Identification, Evolutionary Expansion, and Expression Analyses of Aux/IAA Gene Family in Castanea mollissima During Seed Kernel Development
Source: Biology (Basel). 2025 Jul 3;14(7):806. doi: 10.3390/biology14070806 (PMC12292723; doi:10.3390/biology14070806)

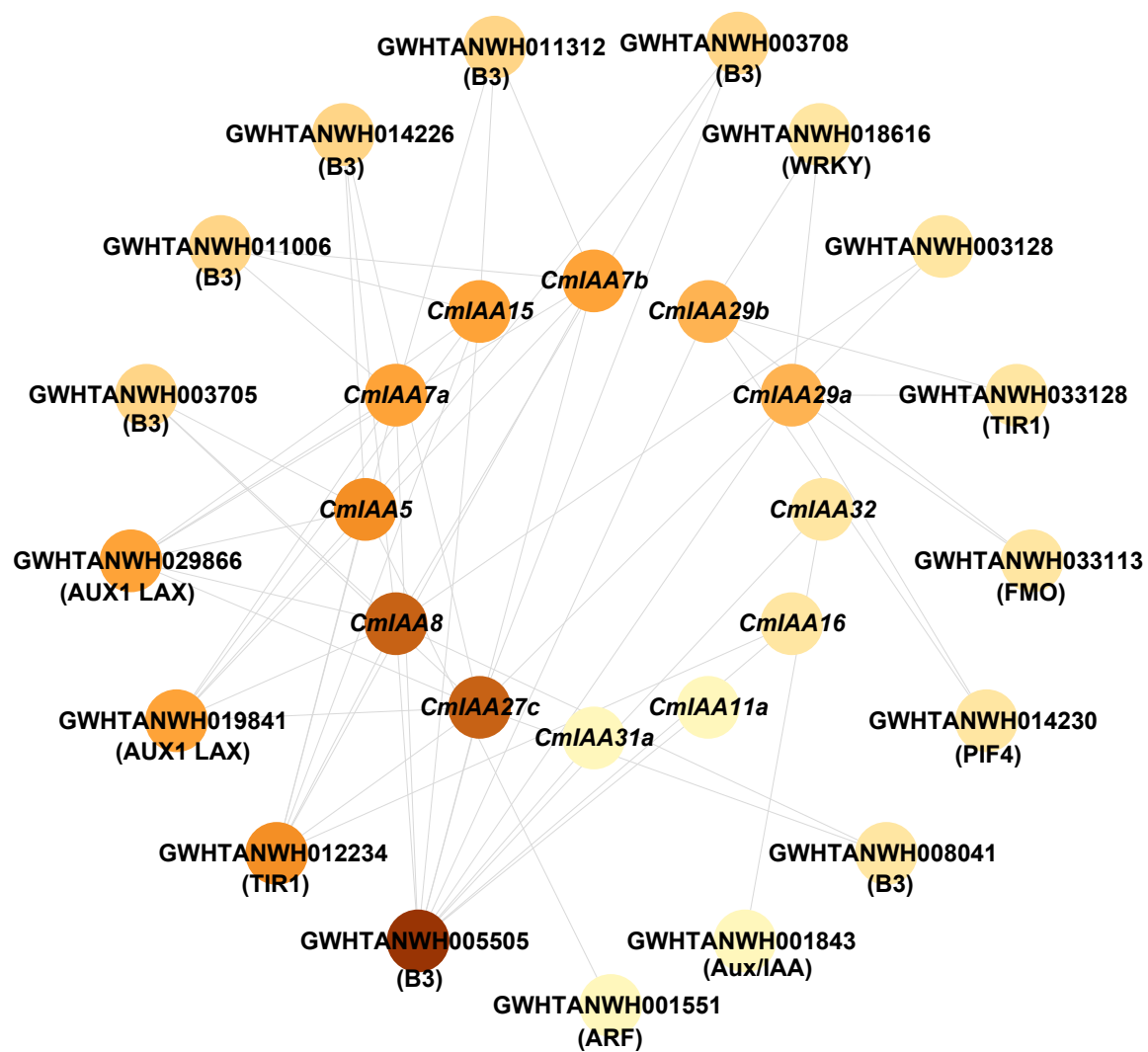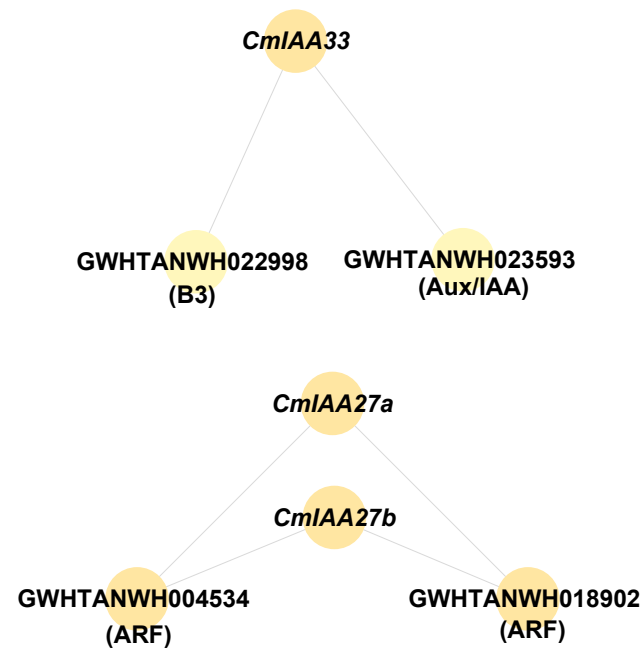

Supplement: Supplementary file 1 [file biology-14-00806-s001.zip › Figure S1 CmAuxIAA protein interaction network analysis.txt.pdf]

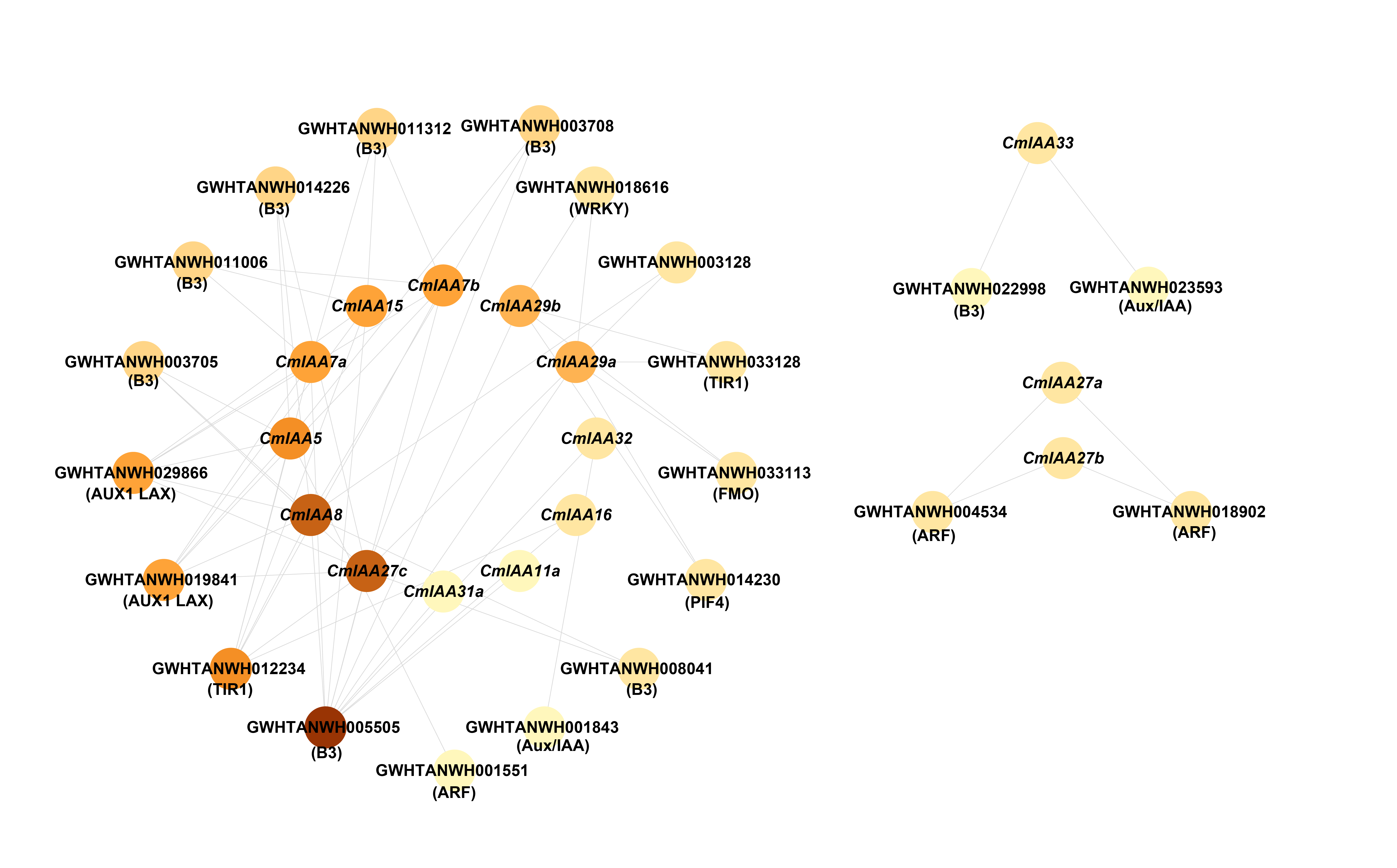

Supplement: Supplementary file 1 [file biology-14-00806-s001.zip › Figure S1 CmAuxIAA protein interaction network analysis.txt.png]

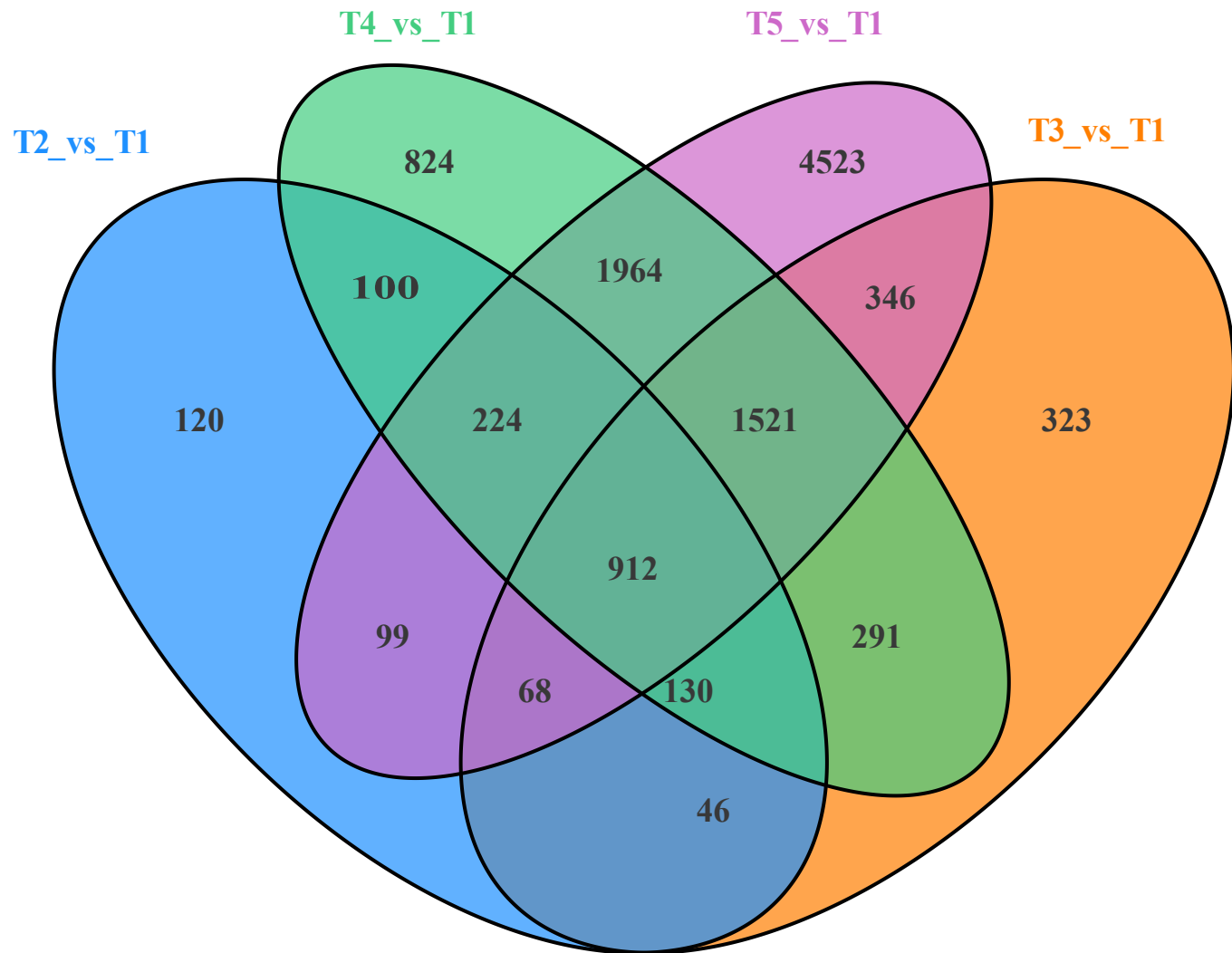

Supplement: Supplementary file 1 [file biology-14-00806-s001.zip › Figure S2 Venn diagram of DEGs in T1 stage and the other four (T2-T5) stages.pdf]

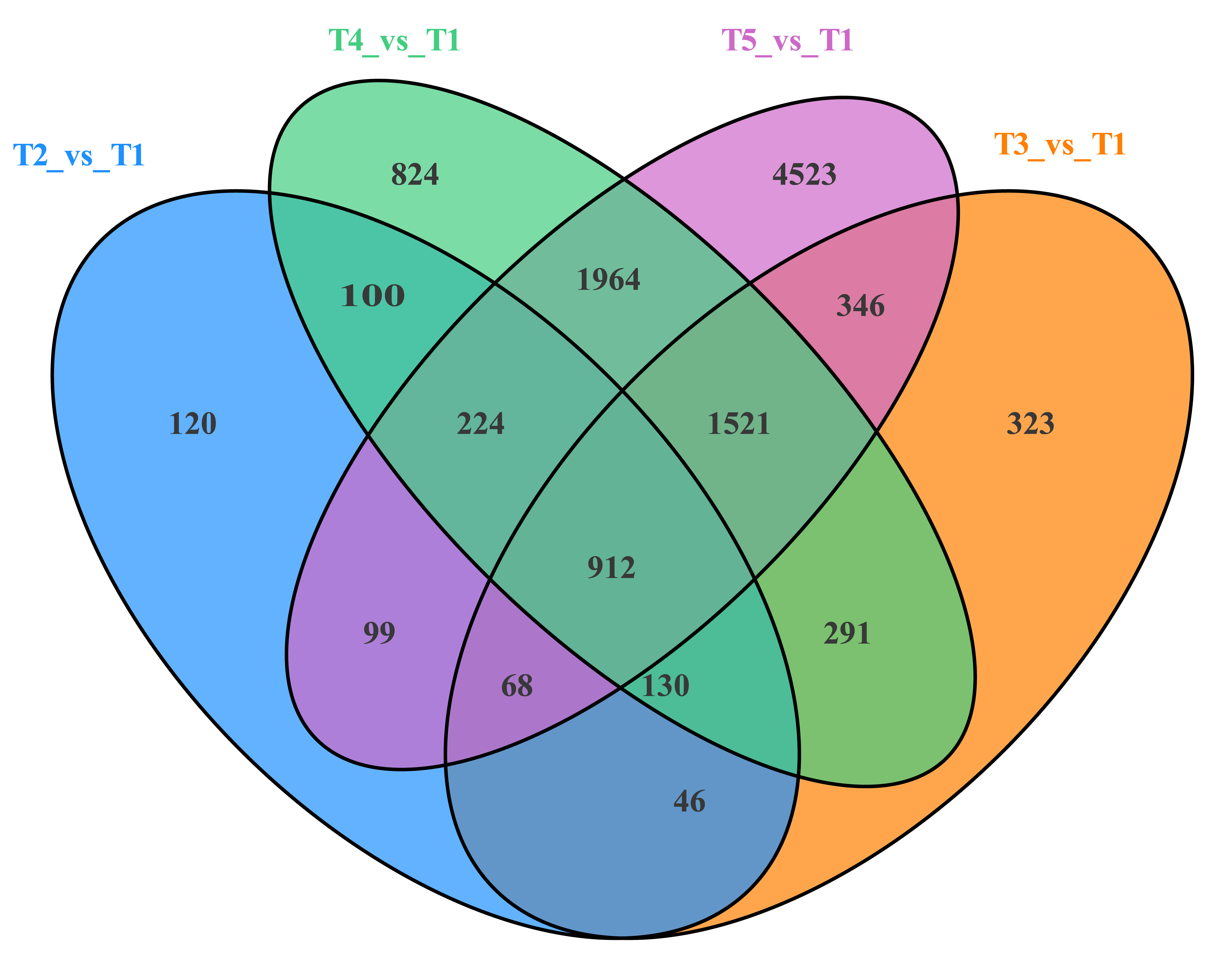

Supplement: Supplementary file 1 [file biology-14-00806-s001.zip › Figure S2 Venn diagram of DEGs in T1 stage and the other four (T2-T5) stages.png]

Cluster Dendrogram

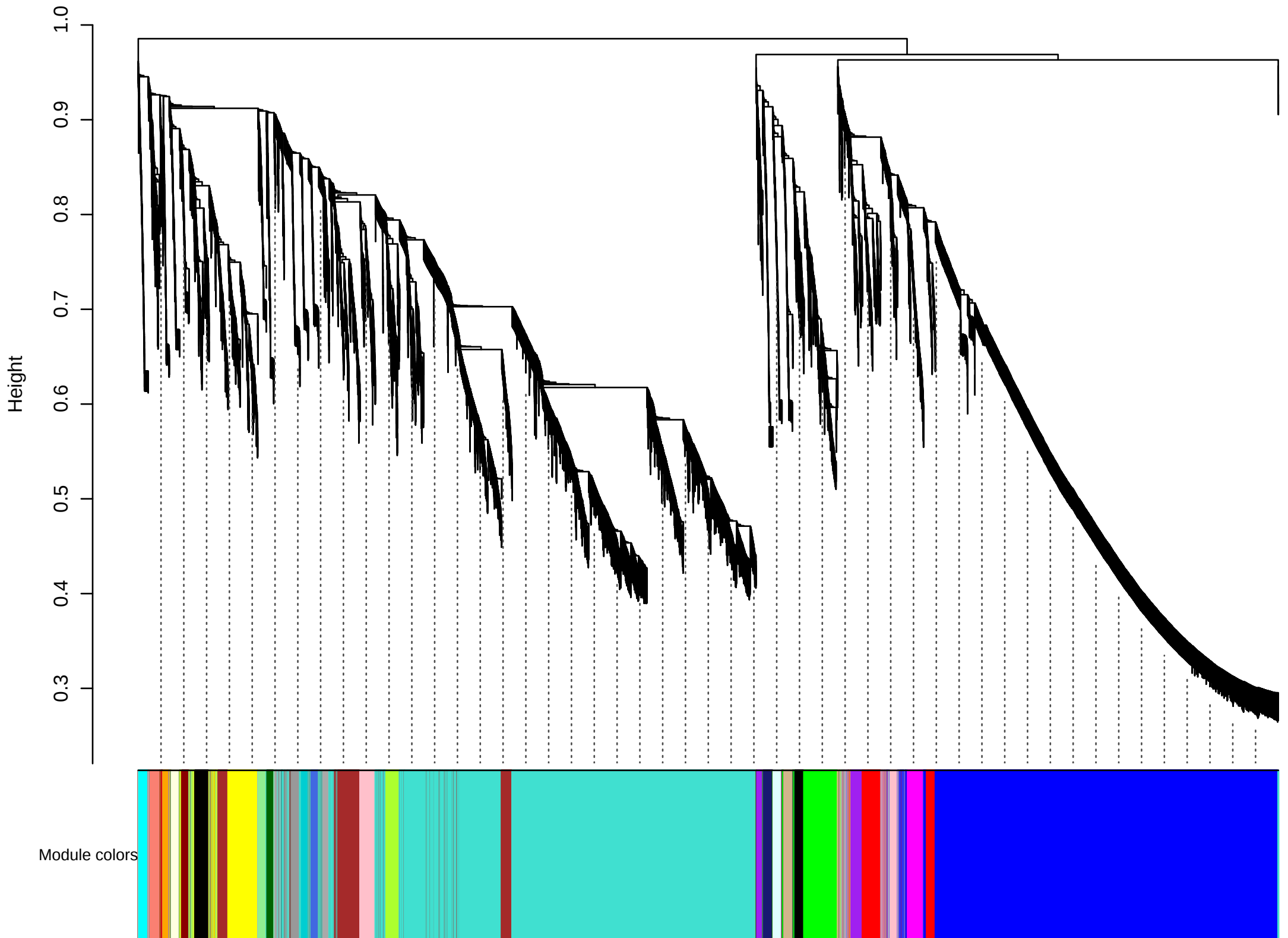

Supplement: Supplementary file 1 [file biology-14-00806-s001.zip › Figure S3 The clustering dendrogram of genes identifying the WGCNA modules.pdf]

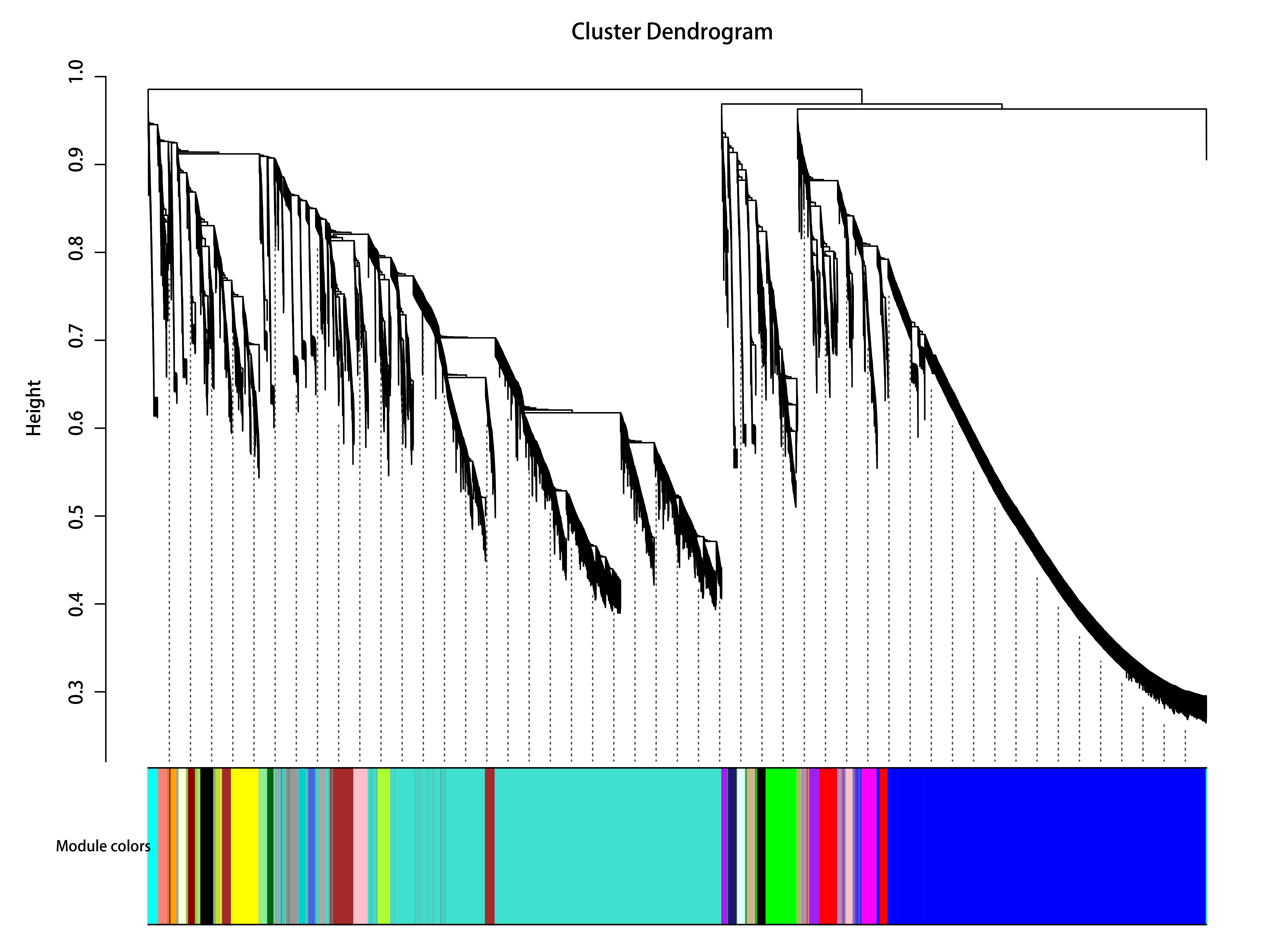

Supplement: Supplementary file 1 [file biology-14-00806-s001.zip › Figure S3 The clustering dendrogram of genes identifying the WGCNA modules.png]
